# A Wirelessly Powered Smart Contact Lens with Reconfigurable Wide Range and Tunable Sensitivity Sensor Readout Circuitry

**DOI:** 10.3390/s17010108

**Published:** 2017-01-07

**Authors:** Jin-Chern Chiou, Shun-Hsi Hsu, Yu-Chieh Huang, Guan-Ting Yeh, Wei-Ting Liou, Cheng-Kai Kuei

**Affiliations:** 1 Department of Electrical and Computer Engineering, National Chiao-Tung University, Room 617, Engineering Building 5, No. 1001, Ta Hsueh Rd., Hsinchu 30010, Taiwan; chiou@mail.nctu.edu.tw; 2 Institute of Electrical Control Engineering, National Chiao-Tung University, Room 617, Engineering Building 5, No. 1001, Ta Hsueh Rd., Hsinchu 30010, Taiwan; yuchieh.ece99g@g2.nctu.edu.tw (Y.-C.H.); x210469@nctu.edu.tw (G.-T.Y.); steven61617@yahoo.com.tw (W.-T.L.); gloooomylife@gmail.com (C.-K.K.)

**Keywords:** capacitance-to-digital converter (CDC), capacitive sensor, soft contact lens, UHF RFID Class1 Gen2

## Abstract

This study presented a wireless smart contact lens system that was composed of a reconfigurable capacitive sensor interface circuitry and wirelessly powered radio-frequency identification (RFID) addressable system for sensor control and data communication. In order to improve compliance and reduce user discomfort, a capacitive sensor was embedded on a soft contact lens of 200 μm thickness using commercially available bio-compatible lens material and a standard manufacturing process. The results indicated that the reconfigurable sensor interface achieved sensitivity and baseline tuning up to 120 pF while consuming only 110 μW power. The range and sensitivity tuning of the readout circuitry ensured a reliable operation with respect to sensor fabrication variations and independent calibration of the sensor baseline for individuals. The on-chip voltage scaling allowed the further extension of the detection range and prevented the implementation of large on-chip elements. The on-lens system enabled the detection of capacitive variation caused by pressure changes in the range of 2.25 to 30 mmHg and hydration level variation from a distance of 1 cm using incident power from an RFID reader at 26.5 dBm.

## 1. Introduction

An increase in on-lens sensors presents an attractive proposition to maximize the procurement of eye health information to the maximum possible extent. The geometry and size constraints of a contact lens necessitate components that are miniature and compact to fit in the lens. Hence, a wirelessly powered smart contact lens (SCL) system with a reconfigurable capacitance-to-digital converter (CDC) was proposed in this study. The proposed soft contact lens was fabricated with hydroxyethyl methacrylate (HEMA) hydrogel and a commercial manufacturing process to improve edge configuration, compliance, and comfort [1]. Intraocular pressure (IOP) measurement for glaucoma prevention was used to illustrate the functionality of the proposed SCL system in the study.

Continuous IOP monitoring in glaucoma patients with high accuracy and high reliability is constantly required to enable prompt detection and daily tracking [2]. Methods were proposed for continuous and long-term IOP monitoring by using an implant pressure sensor [3,4,5] and eye curvature monitoring [6]. Pressure sensor implants are invasive and require surgery, thereby reducing patient compliance. Additionally, the implants usually require high incident wireless power to operate the devices, and this could cause a rise in the temperature of eye tissues. In contrast, the on-lens eye curvature method is less invasive and can provide direct access to the eye surface with minimal disturbances. However, the sensors are typically fabricated on hard silicone or plastic materials [6], and thus lead to discomfort in terms of long-term wear.

Previous studies demonstrated a microelectromechanical system (MEMS) capacitive pressure sensor on a contact lens [7,8] and sensor chips [9,10] for long-term IOP monitoring. An extant study demonstrated the wireless harvesting of smart contact lens and antenna design [10]. This study involved proposing a reconfigurable CDC to improve the capacitance detection characteristics and demonstrated the functionalities of a wirelessly powered SCL system. The results indicated improvements in the capacitance detection range and sensitivity when compared with those of the CDC as reported in previous studies [9,10]. The rest of the paper is organized as follows: Section 2 describes published research that includes studies examining system design, antennae, sensor chips, and capacitive sensors; Section 3 describes the improved sensor readout circuitry and contact lens assembly process; Section 4 discusses the results of experimental investigations of the wirelessly operated SCL system. Finally, conclusions and discussions are presented in Section 5.

## 2. Related Work

This section briefly summarizes extant research including system design, antennae, sensor chips, and capacitive sensors.

### 2.1. System and Sensor Chip Design

**Figure 1:**
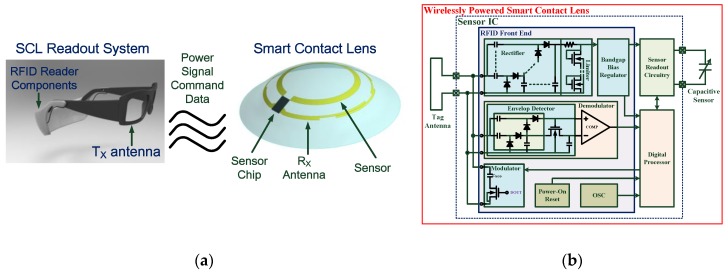
(**a**) Proposed smart contact lens (SCL) system architecture; (**b**) Block diagram of the proposed smart contact lens.

A previous study [9] presented an integrated wireless SCL system as shown in Figure 1a,b. The SCL system included a capacitive sensor for eye healthcare monitoring, a silicon chip with readout circuitry and an ultra-high frequency (UHF) radio-frequency identification (RFID) Gen-2 wireless transceiver, and a small loop antenna. The SCL system communicated with an on-glass RFID reader for sensing data collection and sensor control.

Figure 1b shows the architecture of a proposed sensor chip. The sensor chip includes the analog front-end circuits for power scavenging and data demodulation, a digital processor for protocol format generation and sensing information processing, and a sensor readout circuitry to acquire the sensor information. A capacitive sensor was employed in the design to detect eye curvature changes due to IOP deviations. The measured capacitance value was converted to a bit-stream format for further digital processing. Size constraints on the lens led to low antenna efficiency and low energy storage capability and limited the available power on the lens. Therefore, low power electronics design and efficient power management were crucial in the proposed system. Furthermore, separated regulators were used in the design to avoid noise coupling between digital and analog circuits, that is, the analog supply voltage for sensitive sensor readout circuitry and the low-voltage supply for digital circuits. In order to evaluate the radio frequency (RF) front end performance, the chip was measured using standard RFID testing equipment (CISC RFID Xplorer) [11] that allowed programming instructions to be sent to the RFID tags using standard RFID commands or user-defined commands by following the protocol definition. A continuous wave signal from the CISC RFID Xplorer was directly connected to the RF input of the proposed sensor chip for measurement purposes. The rectifier with an RF input level of 1.74 mW (2.4 dBm) had a power conversion efficiency (PCE) of 11.2% and produced 3.13 V across a 50-kΩ load, which was sufficient for circuit operations of the proposed sensor chip. Detailed descriptions of blocks and measurements could be found in previous studies [9,10].

### 2.2. Wireless Energy Harvesting and Antenna Design

**Figure 2:**
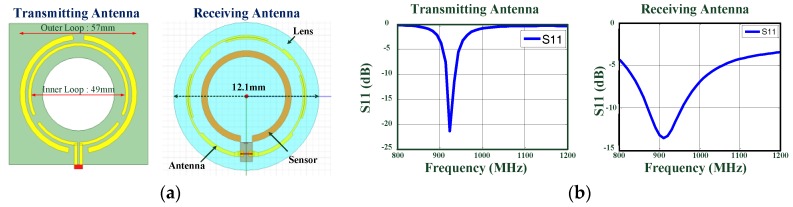
(**a**) Prototypes of transmitting (left) and receiving antennas (right); (**b**) Simulated |*S*_11_| of transmitting antenna (left) and receiving antenna (right).

In addition to the low power consumption requirement of sensor chips, limiting the energy source needed to sustain long-term sensing is another critical challenge for a wirelessly powered SCL system. It was challenging to supply the on-lens device with sufficient energy over an extended lifetime [12]. The sensor chip was wirelessly powered by an external electromagnetic (EM) energy source to avoid the need for batteries on the lens. It was also necessary to minimize the power consumption of the wireless monitoring system to sustain long-term bio-signal acquisition using low RF power. In conjunction with path and tissue losses, the relative location of the power transmitter (on the glasses) and receiver (on the lens) affects the amount of power received, especially for highly oriented small-size antennae. Thus, ring-type geometry was used in the sensor and antenna design due to the need to restrict the area of the components to avoid vision blockage. It was also necessary for the lens thickness to be close to that of commercial products to reduce contact lens discomfort [1]. This makes it difficult to locate matching and tuning components on the lens. A single-turn loop receiving antenna for UHF operation was implemented on the contact lens subject to these constraints. Impedance matching between the RFID sensor chip and antenna was achieved using the antenna profile by changing the segment length of the two wires and the gap spacing between them. Figure 2a,b shows the prototypes and simulation results of the transmitting and receiving antennas, respectively. In order to further improve power transfer, a segmented antenna with a gap between the two loops was used to create a uniform magnetic field distribution [13]. The uniform field distribution of the proposed transmitting antenna could enhance energy transmission and provide robust operation with respect to shifts in the relative location and alignment of the contact lens and the glass caused by eye movement and blinking.

**Figure 3:**
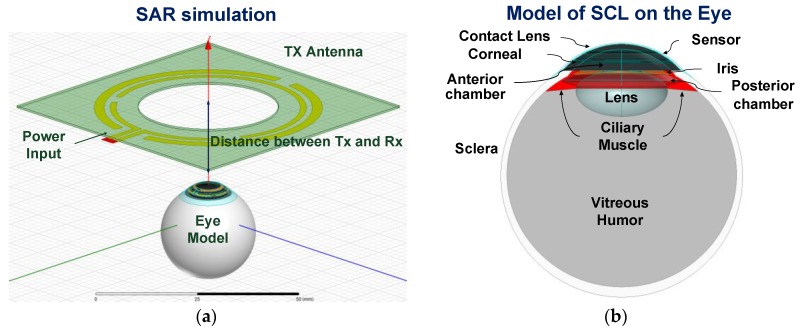
(**a**) Configuration of the simulation; (**b**) Eye model for specific absorption rate (SAR) simulation.

For safety reasons, it was necessary to maintain the increase in eye temperature under RF exposure to less than 1 °C [14]. An eye model with seven tissues [15], as shown in Figure 3a,b, was used to investigate the tissue influence and the specific absorption rate (*SAR*). The temperature increase in the eye tissue under short-term EM exposure can be obtained by a linear approximation of the bio-heat equation [14] as follows:

(1) $$SAR=c\frac{\Delta T}{\Delta t}$$

where *c* denotes the heat capacity of the tissue, Δ*T* denotes the temperature change, and Δ*t* denotes the exposure time. The maximum increase in temperature following 1 s of 30 dBm EM exposure corresponded to only 0.008 °C at 10 mm and 0.002 °C at 20 mm. A previous study discussed and summarized wireless energy harvesting, antenna design, and impact on eye tissues [10].

### 2.3. Capacitive Sensor Design

**Figure 4:**
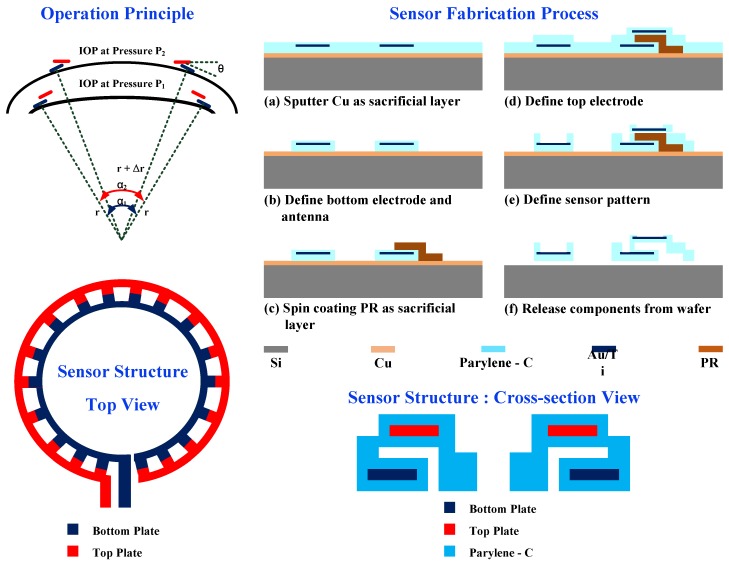
Principle and fabrication process of the capacitive pressure sensor.

Extant studies [7,8] presented the design and fabrication of an IOP capacitive sensor. Figure 4 shows the principle and fabrication process of the IOP capacitive sensor. The contact lens was placed tightly on the surface of the cornea, and thus the distance between the on-lens electrodes followed the curvature changes due to the IOP changes (from P_1_ to P_2_). The total capacitance is derived as follows:

(2) $$C\left(\theta \left(\alpha \right)\right)=N\frac{\varepsilon_{r}\varepsilon_{0}w}{\tan\theta }\ln\frac{d-l\tan\theta +l\sin\theta }{d-l\tan\theta },\;\theta \left(\alpha \right)≅\frac{\frac{\pi }{2}\left(\Delta r+d\right)+\alpha r}{r+\Delta r+d}$$

where *N* denotes the number (18) of parallel capacitors, *d* denotes the gap distance (10 µm), *α* denotes the angle between two ends of the capacitive sensor, and *w* (400 µm) and *l* (100 µm) denote the unit width and unit length of a unit capacitor, respectively. A micro capacitive sensor using top-down parallel plates was fabricated using MEMS methods on the HEMA substrate. The fabrication process is described as follows. First, a sacrificial copper (Cu) layer was sputtered on the Parylene-C substrate. The areas of the bottom capacitor electrodes and the loop antenna were formed by a lift-off process with titanium (Ti; 600 Å) and gold (Au; 3500 Å) and defined by a reactive-ion etching mask. The insulating layer was implemented with Parylene-C with 1 µm thickness. The upper electrode was also formed with Titanium (Ti; 600 Å) and gold (Au; 3500 Å), which were defined after the photolithography and the lift-off process. Finally, the Cu sacrificial layer and the sacrificial layer between the parallel metal layers were removed. The sensor was placed on the inner area, and the antenna was placed outside a contact lens to maximize the effective area and efficiency.

In order to verify the characteristics of the capacitor sensor, the ex vivo porcine eye connected to a syringe pump was filled with increasing and decreasing amounts of water to generate periodical intraocular pressure variations between 0 mmHg and 4.5 mmHg for measurement. The measurements indicated that the sensitivity of the fabricated capacitive sensor corresponded to 4.4 fF/mmHg [8]. To compensate for the capacitance variation of the on-lens sensor owing to dielectric variation and various wearers’ eye conditions, the required capacitance detection range of sensor the readout circuitry was extended to 120 pF [7,8].

## 3. Materials and Methods

The SCL system included a capacitive sensor for eye healthcare monitoring, a silicon chip with readout circuitry and a UHF RFID Gen-2 wireless transceiver, and a small loop antenna. A reconfigurable capacitive sensor readout circuitry that occupied the same chip size as that used in a previous study [10] was employed in the design to overcome sensor variations during the fabrication and baseline drift of various wearers’ eye conditions. The sensor chip of our on-lens system is an integrated single chip solution dedicated to smart contact lens applications; and the chip size and geometrical dimensions were more optimized as compared with those of reported works [16,17,18,19]. The SCL system communicated with an on-glass RFID reader for sensing data collection, sensor control, and calibration for individuals. Furthermore, the on-lens system used commercially available bio-compatible HEMA hydrogel with a standard thickness of 200 μm and a commercial manufacturing process to reduce discomfort and increase compliance.

### 3.1. Design of the Sensor Readout Circuitry

Capacitive sensor interfaces are widely used when compared with resistive sensors due to inherent energy benefits and the absence of static current requirements [20,21]. The reconfigurable CDC proposed in this study provided moderate resolution, low power consumption, and a small chip area. It also simultaneously ensured the maximization of detection sensitivity as well detection range, and thus the proposed CDC was extremely suitable for the on-lens system in the study when compared with the CDCs reported in previous studies [20,21,22,23].

**Figure 5:**
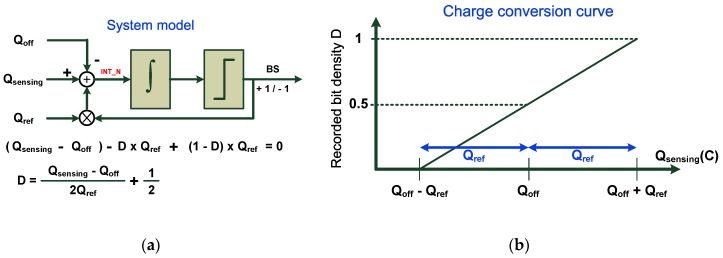
(**a**) System model of the capacitance-to-digital converter (CDC); (**b**) Charge conversion curve of the CDC.

In order to improve the linearity and resolution of the readout circuitry, a 1-bit delta-sigma (Δ-Σ) modulator was adopted to convert the measured sensor value to a digital bit stream. The recorded bit density represented the relationship between the on-chip reference and the sensor value under the test. The converted digital bit stream was fed to a digital counter, and a decimation filter was used to remove undesired high-frequency fluctuations and out-of-band noise. The system model is shown in Figure 5a, and a charge balancing process was used to illustrate the converter operation [20]. The negative feedback in the modulator ensured charge balance and resulted in a zero average charge flowing into the loop filter [20] as follows:

(3) $$\left(Q_{\mathrm{sensing}}-Q_{\mathrm{off}}\right)-D\times Q_{\mathrm{ref}}+\left(1-D\right)\times Q_{\mathrm{ref}}=0$$

where *D* denotes the bit density of the output bit stream BS (0 ≤ *D* ≤ 1). Solving for *D* results in the following expression:

(4) $$D=\frac{Q_{\mathrm{sensing}}-Q_{\mathrm{off}}}{2\times Q_{\mathrm{ref}}}+\frac{1}{2}$$

which corresponds to the desired representation of the sensing charge *Q*_sensing_. The center of the charge conversion curve is denoted by *Q*_off_, and the offset charge (denoted as *Q*_off_) provides flexibility for baseline tuning. The sensitivity of conversion was inversely proportional to *Q_ref_*, and the reference charge *Q*_ref_ provided sensitivity tuning and a detection range of 2*Q*_ref_. The charge conversion curve is summarized in Figure 5b.

**Figure 6:**
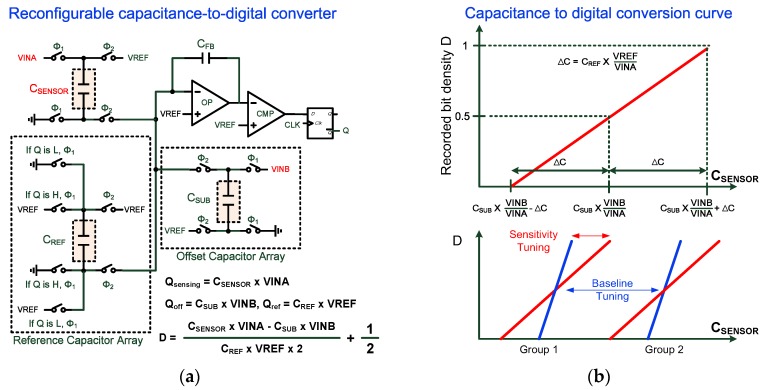
(**a**) A schematic of the proposed reconfigurable CDC; (**b**) Conversion curve and tuning behaviors of CDC.

The proposed reconfigurable CDC as shown in Figure 6a consisted of several capacitance arrays, an integrator, a comparator, an analog signal generator, and digital control circuits. For capacitive sensing, *Q*_sensing_ = *VINA* × *C*_SENSOR_, *Q*_off_ = *VINB* × *C*_SUB_, and *Q*_REF_ = *VREF* × *C*_REF_. The capacitance detection range of the proposed CDC is as follows:

(5) $$C_{\mathrm{SUB}}\times \frac{VINB}{VINA}-C_{\mathrm{REF}}\times \frac{VREF}{VINA}\leq C_{\mathrm{SENSOR}}\leq C_{\mathrm{SUB}}\times \frac{VINB}{VINA}+C_{\mathrm{REF}}\times \frac{VREF}{VINA}$$

where *VREF*, *VINA*, and *VINB* denote the on-chip scalable voltage references, *C*_REF_ denotes the reference capacitor, and *C*_SUB_ denotes the offset capacitor. Additionally, *C*_SUB_ is used for coarsely tuning the capacitor range, and *C*_REF_ is applied for sensitivity tuning of the conversion gain. Voltage scaling of *VINB*/*VINA* was used to further extend the capacitance detection range and prevented the implementation of large on-chip capacitors. The conversion behaviors of the proposed reconfigurable CDC are summarized in Figure 6b. The capacitor arrays and voltage references are wirelessly controlled by the commands from the on-glass RFID reader, and the maximum sensitivity and wide detection range could be simultaneously achieved with this topology.

### 3.2. Contact Lens Assembly Process

**Figure 7:**
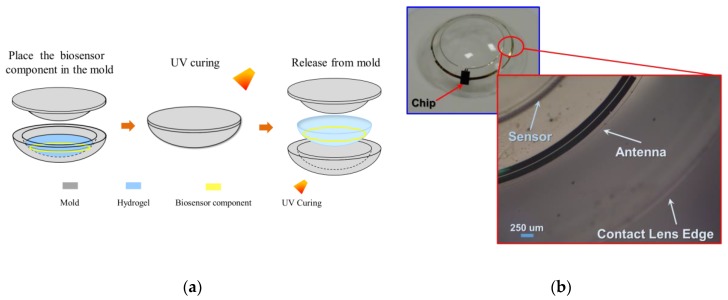
(**a**) Contact lens assembly process; (**b**) Assembled wrinkle free soft contact lens.

A general cast molding method was used to integrate the chip, receiving antenna, and biosensor on a soft contact lens with commercially available biocompatible materials. In order to reduce contact lens discomfort and increase compliance, it was necessary to fit the design of the contact lens to a standard (normal/universal/general) contact lens specification [1] involving thicknesses of 200 μm and 100 um at the peripheral and optical areas, respectively, of a contact lens. In this study, a standard contact lens fabrication process was utilized for the proposed smart contact lens packaging with hydrogel-based material. Figure 7a shows the process flow of the hydrogel-based contact lens biosensor system package with a pair of convex and concave contact lens molds. In the process, the proposed biosensor component was placed on a concave mold, and its center was aligned with the center of the mold. This was followed by pouring a hydrogel material into a concave mold and then combining the convex and concave molds. The contact lens with the molds was then cured with ultraviolet (UV) light. Finally, the mold was immersed into a hydration solution to facilitate a hydration reaction in the de-molding process. During the reaction, the contact lenses in the molds absorbed water to become soft and then could easily be detached from the molds. The proposed assembly process was compatible with a standard contact lens process, and it improved the edge smoothness and flatness of the contact lens. The assembly method helped in achieving a wrinkle-free smart contact lens as shown in Figure 7b.

## 4. Results

### 4.1. Sensor Chip Experiments

**Figure 8:**
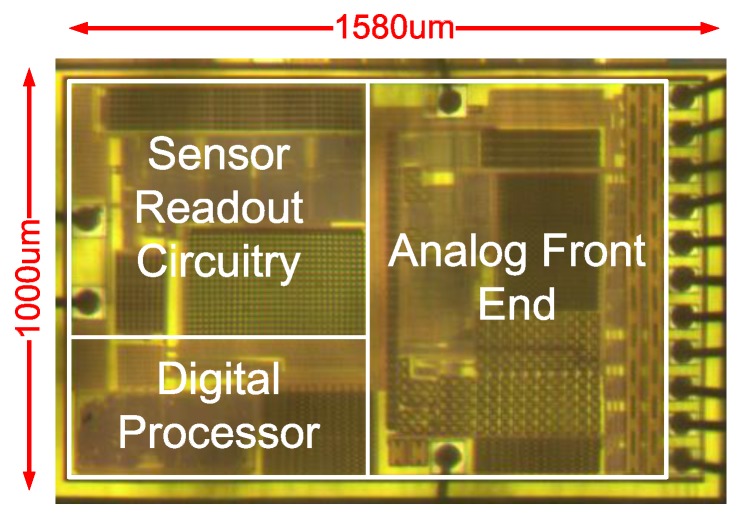
Die photo of the proposed sensor chip.

Figure 8 shows the chip micrograph of the proposed sensor IC. The chip was implemented using 0.18 μm CMOS technology. The dimensions of the chip with input and output pads corresponded to 1 mm × 1.58 mm, and the CDC chip area corresponded to 0.64 mm × 0.64 mm. The proposed CDC topology extended the maximum capacitance detection range from 50 pF to 120 pF within the same chip area when compared with that in a previous study [10]. The sensitivity was improved and could be tuned wirelessly by command programming.

**Figure 9:**
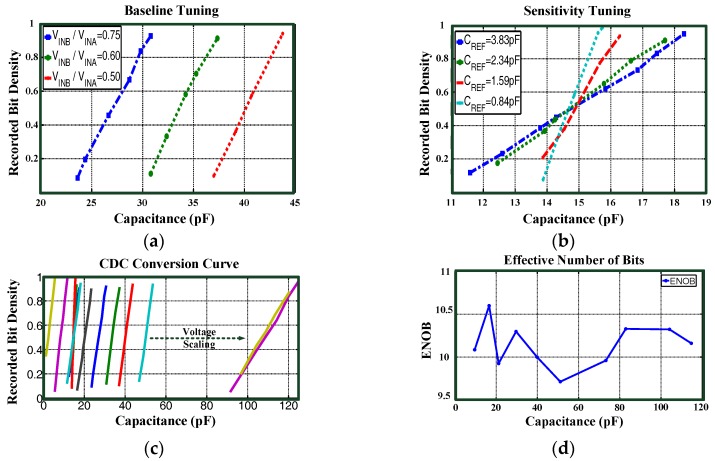
(**a**) Baseline tuning of the CDC; (**b**) Sensitivity tuning of the CDC; (**c**) CDC conversion curves; (**d**) The ENOB of the CDC.

In order to confirm the sensor readout circuitry characteristics, the wired chip bonded on a print circuit board (PCB) was connected to commercial capacitors up to 120 pF for static capacitance conversion testing. The sensor chip was wirelessly powered by 20 dBm incident RF power from the RFID reader at a distance of 2 cm. Different reference capacitance values (denoted as *C*_REF_) were selected for sensitivity tuning, and different voltage scaling ratios VINB/VINA were selected for baseline tuning. In order to evaluate the CDC conversion resolution, the effective number of bits (ENOB) calculation, as defined in a previous study [24], was used. The conversion results are shown in Figure 9. Table 1 shows performance summary and comparison with state-of-the-art CDC.

### 4.2. Wirelessly Powered SCL Expeiments

**Figure 10:**
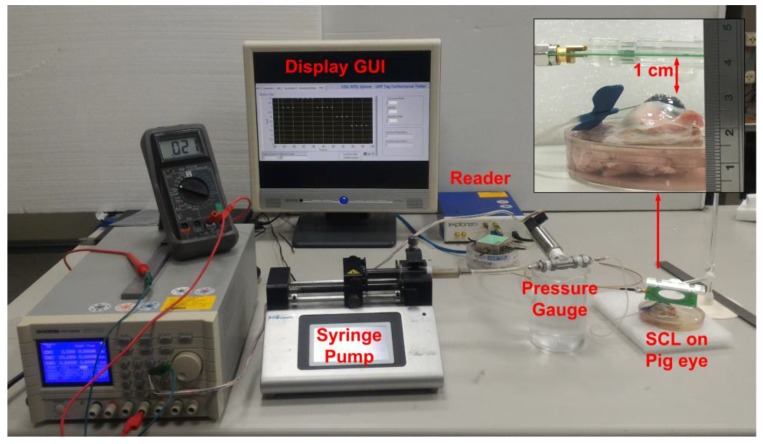
The experimental setup.

The porcine eye (LYD pigs, Guan Dong Market, Hsinchu city, Taiwan, R.O.C) connected to a syringe pump was filled with increasing and decreasing amounts of water to generate periodical intraocular pressure variations in the range of 2.25 and 30 mmHg for measurement purposes. A 20 s pressure variation period was used in the study. The RFID reader was placed at a distance of 1 cm to wirelessly record IOP pressure variation. The photograph of the measurement setup is shown in Figure 10.

**Figure 11:**
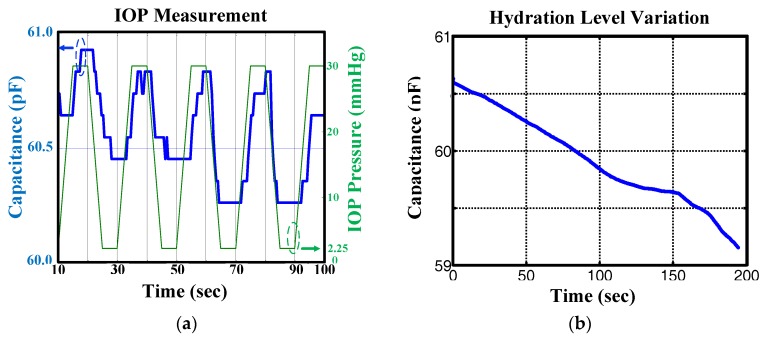
(**a**) Intraocular pressure (IOP) measurement results; (**b**) Hydration level measurement results.

The sensor lens was wirelessly powered by a 26.5 dBm incident RF power from the RFID reader at 920 MHz frequency. Real-time periodical IOP measuring data were directly monitored on a PC and recorded for future clinical study purposes. The recorded periodical IOP pressure variation is shown in Figure 11a. As indicated by the measurement results, the dynamic range of sensor readout circuitry of the proposed sensor lens achieved the IOP range requirement of 2.25–30 mmHg. There is a time delay between the capacitance variation and pressure gauge readings because the IOP is changed by the increasing and decreasing amounts of water. The water was injected and then drained from the pig eye to change the IOP, thus causing a time delay. In addition to IOP measurement, the dielectric capacity increased as the water content increased and vice versa. Figure 11b shows the long-term capacitance change due to the hydration level variation from SCL with the same experiment setup as that shown in Figure 10. The measurements indicated that the proposed SCL could also be used for hydration level detection in the future. Table 2 compares the results with those of state-of-the-art IOP monitoring systems. The proposed design achieved a power consumption of only 110 μW and used RFID addressable communication for sensor control and data communication. With the proposed CDC topology, the maximum sensitivity and wide detection range can be achieved simultaneously. The sensor chip of our on-lens system is an integrated single chip solution dedicated to smart contact lens applications; the chip size and geometrical dimensions were more optimized as compared with those of reported works [3,5,6]. Moreover, the proposed sensor system integrated with a 200-μm thickness contact lens using HEMA hydrogel and standard process can improve discomfort and compliance issues related to contact lenses.

## 5. Discussion and Conclusions

In this study, a wirelessly powered smart contact lens system was proposed for non-invasive and long-term IOP monitoring. The system was composed of an energy transmitter, antennae, and an on-lens sensing device. The proposed SCL system could be wirelessly powered and was able to communicate with commercial Gen2 RFID-compatible equipment to demonstrate periodical IOP pressure variation and hydration level variation measurements. The on-lens system used commercially available bio-compatible HEMA hydrogel with a standard 200-μm thickness and a commercial manufacturing process, and this reduced discomfort and increased compliance. A successful sensor readout distance of 1 cm was achieved under 26.5-dBm incident RF power at a UHF RFID frequency of 920 MHz. The proposed design exhibited low power consumption and was fully integrated onto contact lens material using a standard manufacturing process. The range and sensitivity tuning of reconfigurable readout circuitry provided a reliable operation over sensor fabrication variations. Hydration level detection will be examined in future studies to further extend the application of the proposed SCL system.

## Figures and Tables

**Table 1:** A comparison of the performance of the capacitance-to-digital converter used in the present study to those used in previous studies.

	[20]	[21]	[22]	[23]	This Work
Type	Δ-Σ	Dual-Slope	PWM	Δ-Σ	Δ-Σ
Input Range	0.54–1.06 pF	5.3–30.7 pF	1–6.8 pF	8.4–11.6 pF	1.5–120 pF
Chip Area	0.28 mm^2^	N/A	0.51 mm^2^	2.6 mm^2^	0.41 mm^2^
Power	10.3 μW	110 nW	210 μW	14.9 mW	100 μW
ENOB	12.5 bits	7.05 bits	15 bits	15.3 bits	9.7 bits

**Table 2:** A comparison of the intraocular pressure monitoring system in the present study with those used in previous studies.

References	[3]	[5]	[6]	This Work
Type	Implantable	Implantable	Wearable	Wearable
Communication Technique	Active	Load Modulation	N/A	Load Modulation
Frequency	2.5 GHz	13.56 MHz	27 MHz	920 MHz
Power Consumption	1.4 mW	1.2 mW	N/A	110 μW
Communication Distance	N/A	4 cm	N/A	1 cm
Sensor Type	Capacitive	Resistive	Resistive	Capacitive
Sensor Range	5.3 pF–5.75 pF	5 KΩ–50 KΩ	N/A	1.5 pF–120 pF
ENOB	N/A	8	N/A	9.7
Chip Size	0.5 mm^2^	2 mm^2^	N/A	1.58 mm^2^
Communication Protocol	N.A	ISO-15693	N/A	EPC Class1 Gen2
Lens Thickness	N/A	N/A	400 μm	200 μm
Lens Material	N/A	N/A	Silicone	HEMA
